# Evaluation of Buccal Shelf Bone Dimensions and Bone Density in Adolescents and Adults With Different Mandibular Growth Patterns

**DOI:** 10.7759/cureus.116965

**Published:** 2026-09-26

**Authors:** Nungshinaro Tzudir, Soi Chakraborty, Adeel A Bajjad, Arun Chauhan, Syed Hasnain Nasir, Priyadarshini Ganguly

**Affiliations:** 1 Department of Dentistry, Jain Clinic, Dimapur, IND; 2 Department of Orthodontics and Dentofacial Orthopedics, Kusum Devi Sunderlal Dugar Jain Dental College and Hospital, Kolkata, IND; 3 Department of Orthodontics and Dentofacial Orthopedics, Kothiwal Dental College and Research Centre, Moradabad, IND

**Keywords:** bone density, buccal bone, cone-beam computed tomography, mandible, orthodontic anchorage procedures

## Abstract

Introduction: The mandibular buccal shelf is an important extra-alveolar skeletal anchorage site, but its morphology may vary according to age, skeletal growth pattern, root location, and measurement level. This study evaluated buccal shelf bone thickness, vertical bone height, and bone density in orthodontic patients with different age groups and skeletal growth patterns.

Materials and methods: This retrospective cross-sectional study evaluated cone-beam computed tomography (CBCT) records of 60 skeletal Class I orthodontic patients evaluated between January 2018 and December 2020. Participants were equally distributed among adolescent average, adolescent horizontal, adult average, and adult horizontal growth-pattern groups (n = 15 each) based on mandibular plane angle. Buccal shelf measurements were obtained at the mesial and distal roots of the mandibular first and second molars. Bone thickness was assessed at 6 and 11 mm, vertical bone height at 3, 5, and 7 mm, and bone density at 6 and 11 mm from the cementoenamel junction. Mixed-model analysis of variance followed by Bonferroni-adjusted pairwise comparisons was performed.

Results: Buccal shelf thickness was significantly greater in horizontal than in average growers (mean difference: 1.26 mm; p < 0.001) and in adults than in adolescents (mean difference: 0.32 mm; p < 0.001). Thickness also increased significantly at the 11-mm level and toward the distal root locations. In contrast, vertical bone height was greater on average than in horizontal growers (mean difference: 0.68 mm; p < 0.001) and in adults than in adolescents (mean difference: 0.45 mm; p < 0.001). The age × measurement-level interaction was significant for vertical height (p = 0.015), with a greater adult-adolescent difference at 7 mm than at 3 mm. Bone density showed a significant age × growth-pattern interaction (p = 0.012). The cumulative density was 665 ± 45 Hounsfield units (HU) in adult average growers and 620 ± 40 HU in adult horizontal growers, compared with 568 ± 35 HU and 533 ± 38 HU in adolescent average growers and horizontal growers, respectively.

Conclusion: Mandibular buccal shelf morphology varied significantly with age, skeletal growth pattern, root location, and measurement level. Horizontal growers exhibited greater buccal shelf thickness but lower vertical height, while adults generally demonstrated greater bone dimensions and density. These findings support individualized CBCT assessment when selecting and positioning extra-alveolar skeletal anchorage.

## Introduction

Temporary anchorage devices have become an important component of contemporary orthodontic treatment because they provide reliable anchorage without depending on patient compliance. Miniscrews can facilitate tooth movement in multiple planes and are particularly useful when conventional dental anchorage is insufficient [1]. However, successful miniscrew placement depends on the availability of adequate bone volume and quality at the intended insertion site [2]. The mandibular buccal shelf has emerged as a clinically relevant extra-alveolar site because it can provide substantial cortical bone and allows miniscrews to be placed away from the roots of the mandibular dentition [3].

The mandibular buccal shelf is a region of the alveolar and basal bones located buccal to the mandibular posterior teeth. Its anatomical characteristics are influenced by the morphology and position of the mandibular molar roots, contour of the cortical plate, and spatial relationship between the teeth and surrounding skeletal structures [4]. Adequate buccal bone thickness is essential for achieving the primary mechanical stability of a miniscrew, whereas insufficient bone may increase the risk of cortical perforation, instability, or failure [3]. Similarly, the vertical dimension of the available bone is important when determining a safe insertion site and the appropriate length and angulation of the skeletal anchorage device [5].

The morphology of the mandibular buccal shelf is not consistent throughout the mandible. Differences may occur according to the specific molar and root location, distance from the cementoenamel junction, and level at which the bone is evaluated [4]. Therefore, the first and second mandibular molar regions may contain different amounts of bone. In addition to dimensional characteristics, mineralization and density of the surrounding bone are relevant because cortical bone quality contributes to the mechanical stability of orthodontic miniscrews [3]. A comprehensive evaluation of the buccal shelf should consider both its three-dimensional dimensions and bone density.

The skeletal growth pattern is another important determinant of mandibular morphology. Variations in the vertical relationship of the craniofacial skeleton are associated with differences in mandibular form, alveolar morphology, and spatial arrangement of the posterior teeth. Individuals with horizontal, average, and vertical growth patterns may therefore exhibit differences in the amount and distribution of bone available in the posterior mandibular region [6]. Age-related skeletal maturation may further influence cortical thickness, bone density, and buccal shelf dimensions [7]. Understanding these anatomical variations is particularly relevant when selecting extra-alveolar miniscrew insertion sites for patients with different skeletal characteristics.

Conventional two-dimensional radiographs provide limited information regarding the three-dimensional morphology of the posterior mandible. Cone-beam computed tomography (CBCT) permits multiplanar visualization and quantitative assessment of buccal bone dimensions, root position, and the surrounding osseous structures. Therefore, CBCT-based evaluation can provide more detailed anatomical information for assessing potential skeletal anchorage sites and identifying variations that may not be adequately appreciated on conventional radiographs [8]. Previous investigations have demonstrated that buccal shelf bone thickness and height vary according to the facial growth pattern, age, sex, and anatomical location [4-7]. However, the relationship between skeletal growth patterns and the three-dimensional characteristics of the mandibular buccal shelf remains clinically important because identification of favorable anatomical regions may assist in safer and more individualized miniscrew placement. Therefore, the aim of this study was to evaluate the mandibular buccal shelf bone characteristics in patients with different skeletal growth patterns. The objectives were to compare buccal shelf bone thickness, vertical bone height, and bone density among the different skeletal growth-pattern groups, to assess variations in these parameters at the mandibular first and second molar root locations, and to determine the influence of different measurement levels on these buccal shelf characteristics.

## Materials and methods

Study design and study setting

This retrospective study was conducted in the Department of Orthodontics at Kothiwal Dental College and Research Centre in Moradabad, India. This study was based on the retrospective evaluation of routinely available orthodontic diagnostic records of patients who reported to the department for orthodontic consultation and treatment. Records generated over a three-year period from January 2018 to December 2020 were screened for eligibility. The study did not involve any additional radiographic exposure, as only the previously acquired diagnostic CBCT records were evaluated. The study was initiated after obtaining approval from the Institutional Ethical Review Board of Kothiwal Dental College and Research Centre (approval number: KDCRC/IERB/12/2020/28; approval date: December 26, 2020). Patient confidentiality was maintained throughout the study, and information was removed from the research dataset before analysis. Each eligible record was assigned a unique identification number.

Study population

The study population comprised patients who underwent routine orthodontic diagnostic evaluation during the study period and for whom adequate CBCT and cephalometric records were available. The records were screened sequentially according to the predefined eligibility criteria. Only patients with a skeletal Class I relationship were included in this study. The selected records were subsequently classified into horizontal and average growth-pattern groups according to the mandibular plane angle (Figure 1).

**Figure 1:**
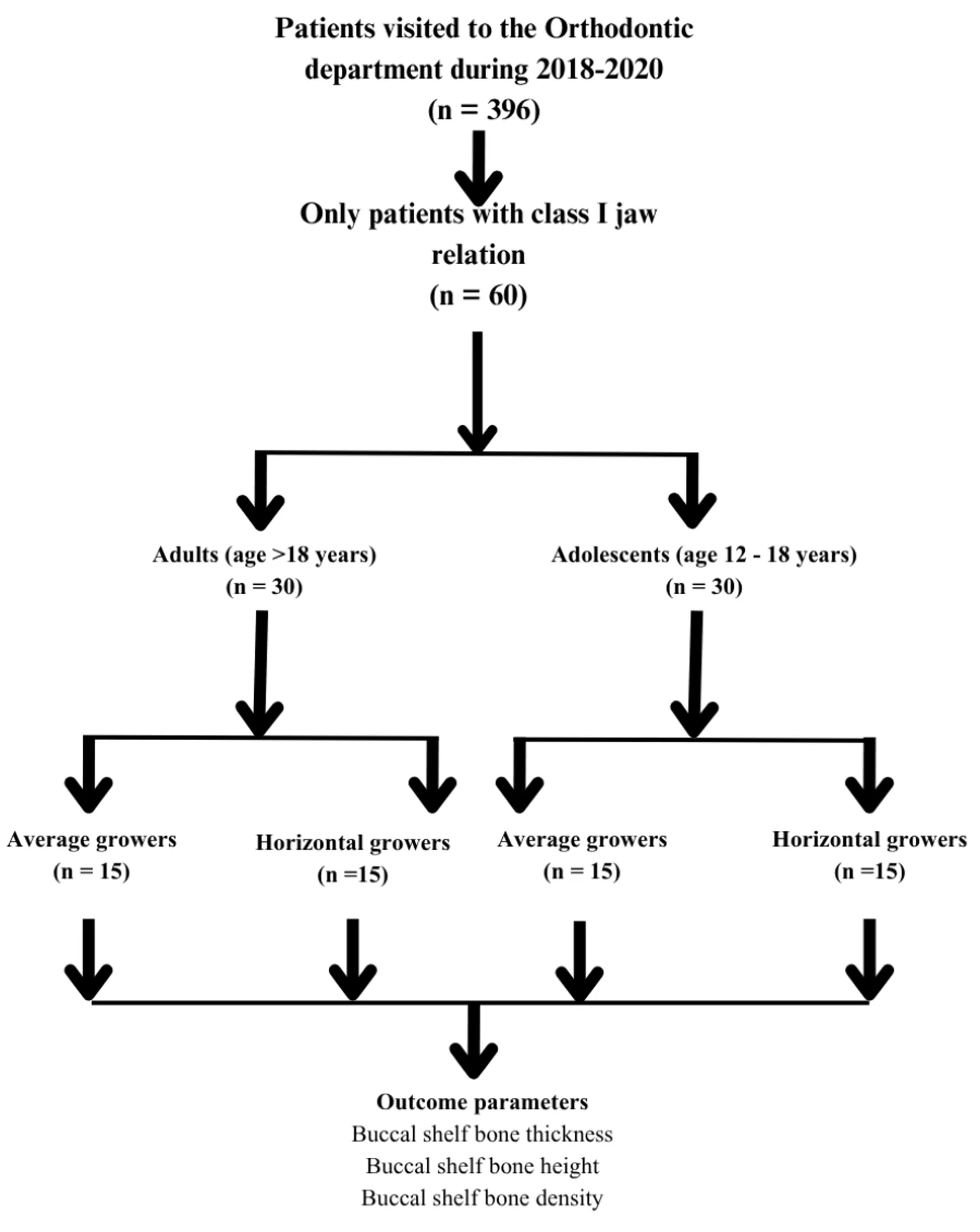
Study flowchart. The figure was manually created by the authors using Canva (Canva Pty Ltd., Surry Hills, Australia).

Eligibility criteria

The inclusion and exclusion criteria applied during the screening of the retrospective records are presented in Table 1.

**Table 1:** Inclusion and exclusion criteria of the study. CBCT: cone-beam computed tomography

Inclusion criteria	Exclusion criteria
Patients aged 12-40 years, who reported to the Department of Orthodontics for routine orthodontic diagnosis and treatment between January 2018 and December 2020.	Patients with a history of previous orthodontic treatment.
Records containing an adequate-quality CBCT scan suitable for the assessment of the mandibular buccal shelf.	Patients with craniofacial syndromes, congenital craniofacial anomalies, or major skeletal deformities.
Complete cephalometric records with clearly identifiable skeletal landmarks.	Patients with marked mandibular or facial asymmetry.
Skeletal Class I relationship, defined by an ANB angle of 0-4°.	Patients with a history of mandibular trauma or previous mandibular surgery.
Availability of clearly identifiable mandibular first and second permanent molars and their roots on CBCT.	Patients with missing mandibular first or second permanent molars or root morphology that prevented standardized measurements.
Adequate visualization of the cementoenamel junction, tooth roots, buccal cortical plate, and surrounding alveolar bone.	CBCT records with severe motion artifacts, distortion, incomplete field of view, or inadequate image quality.
Availability of the mandibular plane angle for the classification of the skeletal growth pattern.	Records with extensive metallic artifacts or other factors obscuring the measurement region.
Records of patients of either sex.	Patients with periodontal bone loss, cysts, tumors, or other pathological lesions involving the mandibular buccal shelf region.

Skeletal classification and growth-pattern assessment

Sagittal skeletal relationship was determined by lateral cephalometric assessment using the ANB angle (the angle between points A and B, which are the deepest points in the anterior concavity of the maxilla and mandible, respectively, and point N, which is the anteriormost point on the frontonasal suture). Records demonstrating an ANB angle between 0° and 4° were classified as skeletal Class I and were eligible for inclusion. Vertical skeletal growth pattern was assessed using the Frankfort-mandibular plane angle (FMA). The FMA was defined as the angle formed between the Frankfort horizontal plane and mandibular plane. The Frankfort horizontal plane was established by joining the porion (the uppermost point on the external auditory meatus) and orbitale (the lowermost point on the bony orbit), whereas the mandibular plane was established by joining the gonion (the constructed point at the intersection of the ramal and mandibular plane) and menton (the lowermost point of bony symphysis).

For subgroup analysis, eligible patients were categorized into adolescent and adult age groups. The adolescent group comprised individuals aged 12-18 years, whereas the adult group comprised individuals aged >18 years at the time of CBCT examination. The cutoff of 18 years was selected to distinguish the adolescent and adult subgroups according to the conventional transition from adolescence to adulthood used in clinical and epidemiological classification. The age at the time of acquisition of the diagnostic CBCT scans was used for classification. After age classification, patients were further categorized according to their vertical skeletal growth patterns using the FMA. For the purpose of growth-pattern classification, patients with an FMA of <25° were classified as horizontal growers, whereas those with an FMA of 25-30° were classified as average growers. These cutoff values were selected based on previously published CBCT investigations evaluating mandibular buccal shelf morphology according to vertical facial growth pattern [9]. Patients with an FMA of >30° were considered to represent a vertical growth pattern and were excluded from the present analysis because an adequate number of eligible records were not available to constitute a sufficiently represented vertical-growth subgroup.

CBCT image acquisition and selection

CBCT examinations included in the study were acquired previously as part of a routine orthodontic diagnostic assessment. No additional CBCT examinations were performed in this study. The original CBCT datasets were retrieved from the institutional archive and assessed for image quality and suitability of the proposed measurements. Only scans with adequate visualization of the mandibular first and second molars and their roots, cementoenamel junction, and buccal cortical plate were included. Records demonstrating substantial motion artifacts, incomplete visualization of the relevant anatomical region, or image distortions that could interfere with the measurement were excluded.

Before measurement, each CBCT volume was reoriented in the multiplanar reconstruction interface using standardized anatomical reference planes. The axial plane was aligned parallel to the mandibular occlusal plane, the sagittal plane was oriented perpendicular to the occlusal plane and aligned with the long axis of the mandibular molar root under assessment, and the coronal plane was oriented perpendicular to the root long axis. The long axis of each mandibular molar root was used as the primary reference for obtaining reproducible measurement sections. The same orientation procedure and anatomical reference points were applied to all CBCT datasets.

CBCT examinations were performed using the Carestream CS 9300 CBCT system (Carestream Dental LLC, Atlanta, GA, USA). The scans were obtained using a 17 × 6 cm field of view (FOV) with a voxel size of 0.20 mm, a tube voltage of 90 kVp, a tube current of 4 mA, and an exposure time of 6.3 seconds. The patients were positioned according to the manufacturer's standard positioning protocol, with the head stabilized to minimize movement during image acquisition. The resulting volumetric datasets were stored in Digital Imaging and Communications in Medicine (DICOM) format. The DICOM datasets were subsequently imported into the three-dimensional imaging software for multiplanar reconstruction.

CBCT image reconstruction and orientation

The images were oriented in the standardized axial, sagittal, and coronal planes before measurements were performed. The long axis of the mandibular molar root and the relevant anatomical reference planes were used to obtain reproducible measurement sections. The same orientation and measurement protocol were applied to all included CBCT datasets. The mandibular first and second molar regions were evaluated separately, and measurements were obtained at the mesial and distal root locations. The anatomical landmarks and reference levels were kept constant throughout the assessment to minimize variations attributable to differences in the image orientation.

Assessment of mandibular buccal shelf dimensions

The mandibular buccal shelf was assessed in the mandibular first and second molars. The principal radiographic parameters were the buccal shelf bone thickness, vertical bone height, and bone density. Measurements were performed at standardized distances from the cementoenamel junction and predefined root locations. The four root locations assessed were the mesial root of the mandibular first molar (M6), the distal root of the mandibular first molar (D6), the mesial root of the mandibular second molar (M7), and the distal root of the mandibular second molar (D7).

Measurement of buccal shelf bone thickness

Buccal shelf bone thickness was measured at two standardized levels, 6 mm and 11 mm, from the cementoenamel junction. A reference line was established along the long axis of the corresponding molar root at each root location. The bone-thickness measurement at each level was then taken horizontally from the buccal root surface to the outer cortical boundary. The measurements were obtained separately for M6, D6, M7, and D7. Thus, each eligible CBCT record provided buccal shelf thickness measurements at four root locations and two assessment levels. Measurements were recorded in millimeters (Figure 2).

**Figure 2:**
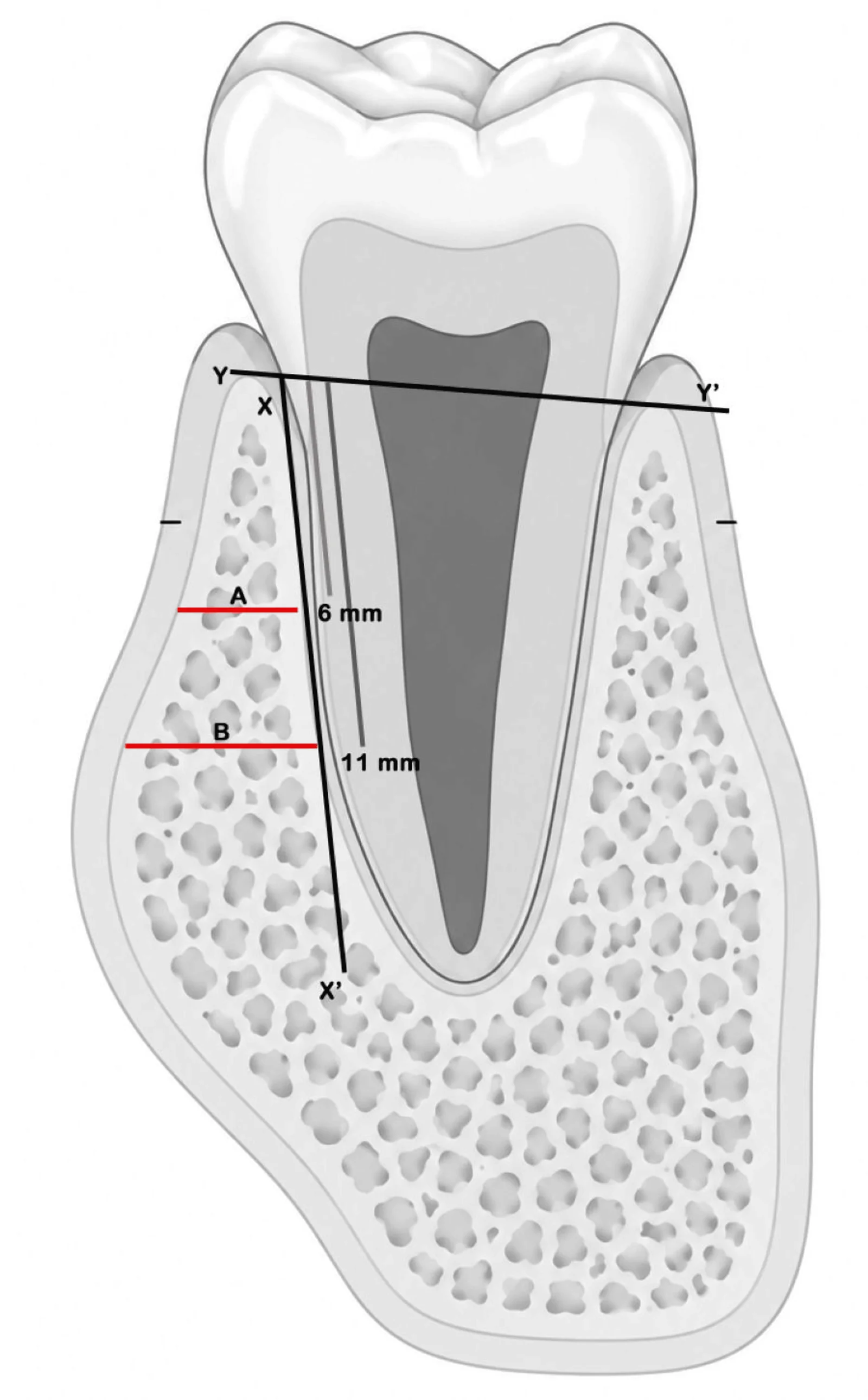
Method of assessing the thickness of the buccal shelf area at different assessment levels. X-X': reference line parallel to the long axis of the molar tooth; Y-Y': reference line from the cementoenamel junction; A: thickness of the buccal shelf bone at 6 mm from the cementoenamel junction; B: thickness of the buccal shelf bone at 11 mm from the cementoenamel junction The figure was manually created by the authors using Canva (Canva Pty Ltd., Surry Hills, Australia).

Measurement of vertical buccal shelf bone height

Vertical buccal shelf bone height was assessed at reference levels of 3, 5, and 7 mm from the cementoenamel junction. At each level, a vertical reference line was established from the cementoenamel junction along the standardized orientation used for the corresponding molar root. The vertical bone height was measured from the specified reference level to the inferior boundary of the available buccal shelf bone along the same vertical measurement line. Measurements were obtained separately at the M6, D6, M7, and D7 root locations and recorded in millimeters (Figure 3).

**Figure 3:**
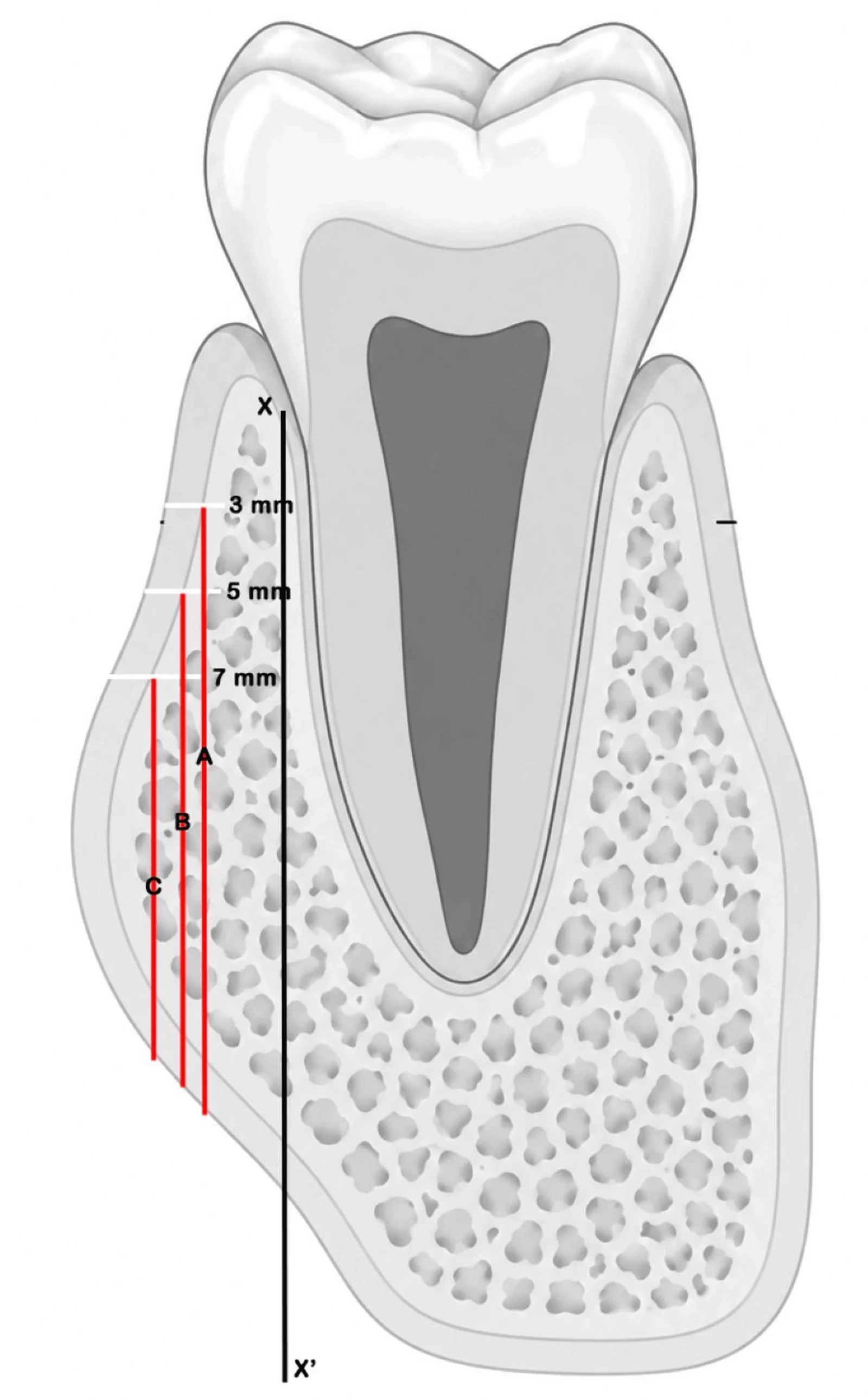
Method of assessing the vertical height of the buccal shelf area at different assessment levels. X-X': vertical reference line from the cementoenamel junction; A: vertical height of the bone at 3 mm from the cementoenamel junction; B: vertical height of the bone at 5 mm from the cementoenamel junction; C: vertical height of the bone at 7 mm from the cementoenamel junction The figure was manually created by the authors using Canva (Canva Pty Ltd., Surry Hills, Australia).

Measurement of buccal shelf bone density

Buccal shelf bone density was assessed at the 6-mm and 11-mm measurement levels at M6, D6, M7, and D7 root locations. Density measurements were obtained from the CBCT dataset using a standardized region of interest (ROI) positioned within the buccal bone while avoiding tooth structure, air spaces, and obvious image artifacts. Density values were recorded in Hounsfield units (HU), as represented in the study dataset. The same image settings and measurement procedures were maintained for all records. Because CBCT-derived density values may be affected by scanner characteristics, acquisition parameters, reconstruction algorithms, and calibration, these values were interpreted primarily for comparison among the groups within the present dataset.

For bone-density assessment, a square ROI measuring 1 × 1 mm was positioned within the buccal cortical bone at the 6-mm and 11-mm reference levels at each M6, D6, M7, and D7 root location. The ROI was positioned immediately buccal to the corresponding root and entirely within the cortical plate, avoiding the tooth structure, periodontal ligament space, cancellous bone, air spaces, and image artifacts. Thus, the density measurement represented cortical buccal shelf bone rather than a mixture of cortical and cancellous bone. The CBCT system underwent routine manufacturer-recommended calibration and quality-control procedures before image acquisition. The same acquisition and reconstruction settings were maintained for all included examinations.

For each participant, cumulative overall density was calculated as the arithmetic mean of the density measurements obtained across all assessed root locations and measurement levels. Thus, the "Cumulative Overall" value represents the overall mean of the individual density measurements included in the analysis. All the measurements were obtained bilaterally from the right and left sides of each patient. For each corresponding root location and measurement level, the right- and left-side measurements were averaged to obtain a single patient-level value. These averaged values were subsequently used for statistical analysis, thereby avoiding the treatment of bilateral measurements as independent observations.

Examiner calibration and measurement reliability

Before commencement of the formal measurements, the examiner was calibrated to identify cephalometric landmarks and CBCT measurement points under the supervision of an experienced orthodontist. A subset of CBCT records was selected for repeated measurements. The measurements were repeated after an interval of approximately two weeks, without reference to the initial measurements. Intra-examiner reliability was assessed using the intraclass correlation coefficient (ICC) based on a two-way mixed-effects model with absolute agreement, and the corresponding 95% confidence intervals (CI) were calculated. The ICC was 0.982 (95% CI: 0.961-0.992) for the ANB angle, 0.987 (95% CI: 0.972-0.995) for the FMA, 0.964 (95% CI: 0.931-0.983) for buccal shelf bone thickness, 0.953 (95% CI: 0.912-0.977) for vertical buccal shelf bone height, and 0.947 (95% CI: 0.901-0.974) for buccal shelf bone density, indicating excellent intra-examiner reproducibility for all measurements.

Sample size and sampling procedure

The sample size was calculated using the G*Power software (Heinrich-Heine-Universität Düsseldorf, Düsseldorf, Germany) through an a priori power analysis based on an independent-group comparison. The effect size was derived from a previous study that evaluated mandibular buccal shelf bone thickness according to facial growth pattern [9]. The analysis was performed with a statistical power of 80% and an alpha error probability of 5%, resulting in a required total sample size of 60 participants. This comprised 30 individuals in each age group (adolescents and adults). Each age group was subsequently stratified into two growth-pattern subgroups, comprising 15 average growers and 15 horizontal growers per age category. The a priori power analysis was based on an independent-group comparison and was used to determine the minimum overall sample size; it was not performed specifically to estimate power for the complete mixed-model analysis of variance (ANOVA) structure involving multiple between-subject and within-subject factors.

Data collection

Demographic and cephalometric data were extracted from the selected orthodontic records. The recorded variables included age at the time of imaging, sex, ANB angle, FMA, and growth-pattern category. The CBCT-derived variables included buccal shelf thickness at 6 and 11 mm, vertical bone height at 3, 5, and 7 mm, and bone density at 6 and 11 mm. Measurements were recorded separately for M6, D6, M7, and D7 root locations. The data were entered into a structured database after radiographic assessment was completed (Figure 4).

**Figure 4:**
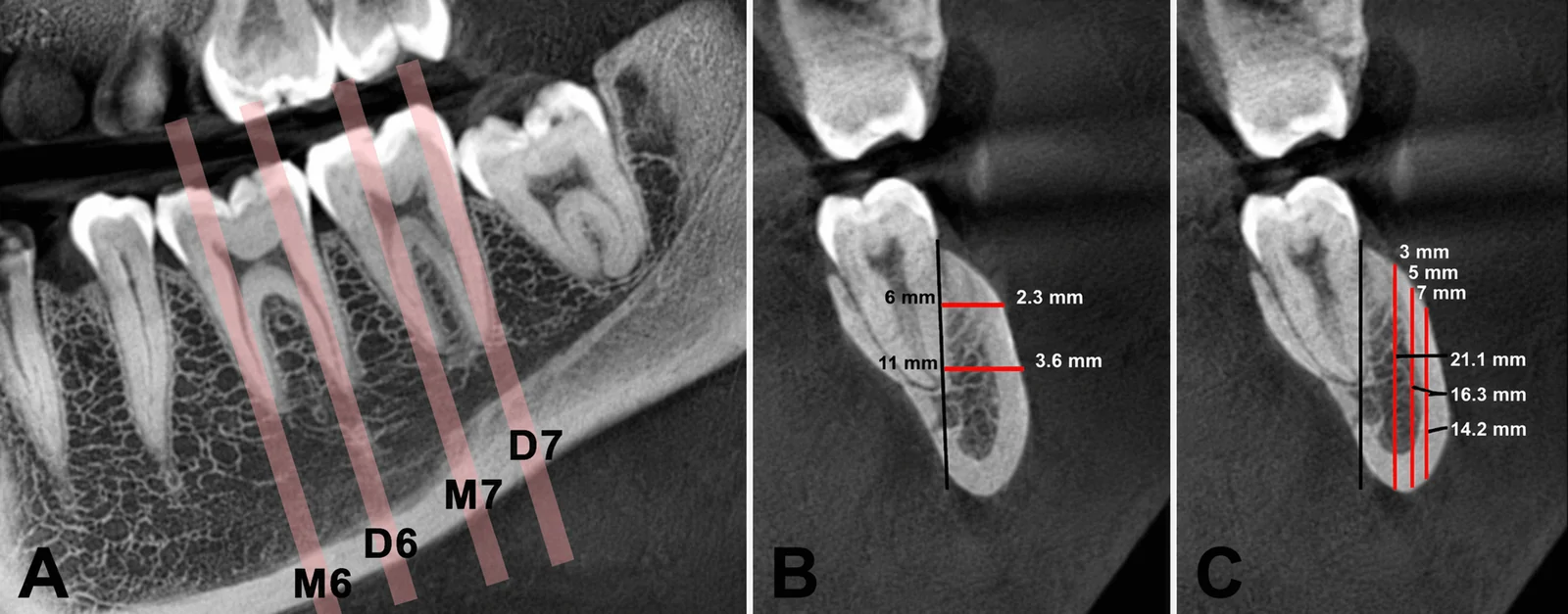
Measurement of buccal shelf area bone thickness and height on cone-beam computed tomographic images of the patient: (A) assessment points at roots of molar teeth in the sagittal view and (B) buccal shelf area bone thickness at 6-mm and 11-mm assessment levels in the axial view and (C) buccal shelf area vertical height of bone at 3-mm, 5-mm, and 7-mm assessment levels in the axial view.

Statistical analysis

Statistical analyses were performed using IBM SPSS Statistics for Windows, V. 26.0 (IBM Corp., Armonk, NY, USA). The distribution of continuous variables was assessed using the Shapiro-Wilk test. Normally distributed continuous variables are expressed as mean ± standard deviation (SD), whereas categorical variables are summarized as frequencies and percentages. The comparability of categorical variables between the study groups, particularly sex distribution, was assessed using the Pearson chi-squared (χ²) test. Differences in continuous baseline variables, including age and cephalometric measurements such as ANB angle and FMA, were assessed using one-way ANOVA when comparing the study groups.

For the primary radiographic outcomes, mixed-model ANOVA was performed to account for repeated measurements obtained from the same individuals. Age group and skeletal growth pattern were considered between-subject factors, whereas root location and measurement level were considered within-subject factors. The model assessed the main effects of age group, growth pattern, root location, and measurement level, together with their relevant interaction effects. The four study subgroups were therefore incorporated within the between-subject component of the mixed-model analysis, while measurements obtained from multiple root locations and reference levels within the same individual were treated as repeated within-subject observations.

When a statistically significant main effect or interaction was identified in the mixed-model ANOVA, Bonferroni-adjusted post hoc pairwise comparisons were performed to determine the specific groups or measurement levels responsible for the observed differences. The magnitude of the ANOVA effects was expressed using partial eta squared (ηp²), whereas Cohen's d was used to quantify the magnitude of differences identified in post hoc pairwise comparisons. Post hoc pairwise comparisons were performed using estimated marginal means derived from the mixed-model ANOVA. Overall marginal means were estimated while accounting for the other factors included in the model, and pairwise comparisons were adjusted using the Bonferroni method. All statistical tests were two-tailed, and statistical significance was set at p < 0.05.

## Results

Study population and baseline characteristics

Sixty eligible orthodontic records were included in the available dataset, with 15 records in each of the four analyzed subgroups. The groups included adolescent average growers, adolescent horizontal growers, adult average growers, and adult horizontal growers. The mean age was 13.4 ± 1.4 years in adolescent average growers, 13.6 ± 1.5 years in adolescent horizontal growers, 19.1 ± 1.6 years in adult average growers, and 19.3 ± 1.7 years in adult horizontal growers (p < 0.001). The sex distribution was balanced, with males comprising eight (53.3%) and females seven (46.7%) in each subgroup (p = 0.992). The horizontal-growth groups demonstrated higher ANB angles and lower FMA than those of the corresponding average-growth groups, confirming the intended skeletal classification (Table 2).

**Table 2:** Distribution and baseline characteristics of the study participants. Data are presented as mean ± SD for continuous variables and n (%) for categorical variables. Age, ANB angle, and FMA were compared using one-way ANOVA, and sex distribution was compared using the Pearson chi-squared (χ²) test. *p < 0.05 was considered statistically significant. ANB: A point-Nasion-B point; FMA: Frankfort-mandibular plane angle; SD: standard deviation; ANOVA: analysis of variance

Variable	Adolescents average (n = 15)	Adolescents horizontal (n = 15)	Adults average (n = 15)	Adults horizontal (n = 15)	Test value	P-value
Age, years, mean ± SD	13.4 ± 1.4	13.6 ± 1.5	19.1 ± 1.6	19.3 ± 1.7	F = 82.4	<0.001*
Sex, male n (%)	8 (53.3%)	7 (46.7%)	8 (53.3%)	7 (46.7%)	χ² = 0.18	0.992
Sex, female n (%)	7 (46.7%)	8 (53.3%)	7 (46.7%)	8 (53.3%)		
ANB angle (°), mean ± SD	2.4 ± 1.1	3.7 ± 1.0	2.5 ± 1.2	3.8 ± 0.9	F = 23.6	<0.001*
FMA (°), mean ± SD	25.3 ± 4.1	20.4 ± 3.9	24.9 ± 4.3	19.9 ± 3.6	F = 21.9	<0.001*

Buccal shelf bone thickness

Buccal shelf bone thickness demonstrated consistent variation according to the growth pattern, age, root location, and measurement level. At both the 6-mm and 11-mm levels, horizontal growers showed greater bone thickness than the average growers. At the 6-mm level, thickness ranged from 3.2 ± 0.5 to 4.2 ± 0.6 mm in horizontal growers compared with 2.1 ± 0.4 to 3.0 ± 0.5 mm in average growers. At the 11-mm level, the corresponding ranges were 4.4 ± 0.6 to 5.7 ± 0.7 mm and 3.1 ± 0.5 to 4.3 ± 0.6 mm, respectively (Table 3).

**Table 3:** Buccal shelf bone thickness according to assessment level and root location. Data are presented as mean ± SD in millimeters. Buccal shelf bone thickness was measured at 6 and 11 mm from the cementoenamel junction at the mesial and distal roots of the mandibular first and second molars. M6: mesial root of the mandibular first molar; D6: distal root of the mandibular first molar; M7: mesial root of the mandibular second molar; D7: distal root of the mandibular second molar; SD: standard deviation

Assessment points	Root level	Adults average, n = 15	Adults horizontal, n = 15	Adolescents average, n = 15	Adolescents horizontal, n = 15
		Mean ± SD	Mean ± SD	Mean ± SD	Mean ± SD
At 6 mm	M6	2.4 ± 0.4	3.5 ± 0.5	2.1 ± 0.4	3.2 ± 0.5
	D6	2.7 ± 0.5	3.8 ± 0.5	2.4 ± 0.4	3.5 ± 0.5
	M7	2.8 ± 0.5	3.9 ± 0.5	2.5 ± 0.4	3.6 ± 0.5
	D7	3.0 ± 0.5	4.2 ± 0.6	2.7 ± 0.5	3.8 ± 0.5
At 11 mm	M6	3.4 ± 0.5	4.8 ± 0.6	3.1 ± 0.5	4.4 ± 0.6
	D6	3.8 ± 0.6	5.2 ± 0.7	3.5 ± 0.5	4.8 ± 0.6
	M7	4.0 ± 0.6	5.4 ± 0.7	3.7 ± 0.5	5.0 ± 0.6
	D7	4.3 ± 0.6	5.7 ± 0.7	3.9 ± 0.6	5.3 ± 0.7

Mixed-model ANOVA demonstrated significant effects of age group (p < 0.001), growth pattern (p < 0.001), root location (p < 0.001), and measurement level (p < 0.001). A significant root × level interaction was also observed (p = 0.008), whereas the age × growth-pattern interaction was not significant (p = 0.152) (Table 4). Because the age × growth-pattern interaction was statistically significant, the effects were further examined using simple-effects comparisons to assess the effect of growth pattern within each age group and the effect of age within each growth-pattern group. These comparisons were interpreted separately rather than relying solely on the corresponding overall main effects.

**Table 4:** Mixed-model analysis of variance for buccal shelf bone thickness. Mixed-model analysis of variance was used, with age group and growth pattern as between-subject factors and root location and assessment level as within-subject factors. *p < 0.05 was considered statistically significant. A: age group; G: growth pattern; R: root location; L: assessment level; SS: sum of squares; df: degrees of freedom; ηp²: partial eta squared

Source of variation	SS	df₁	df₂	F‑value	P-value	ηp²
Between subjects						
Age group (A)	4.8	1	56	25.6	<0.001*	0.314
Growth pattern (G)	18.4	1	56	98.0	<0.001*	0.636
A × G	0.4	1	56	2.1	0.152	0.036
Within subjects						
Root (R)	6.2	3	168	15.4	<0.001*	0.216
Level (L) - (6 vs 11 mm)	8.5	1	56	45.2	<0.001*	0.447
R × L	1.7	3	168	4.2	0.008*	0.070
A × R	0.4	3	168	0.9	0.442	0.016
G × R	0.5	3	168	1.1	0.352	0.019
A × L	0.2	1	56	1.2	0.278	0.021
G × L	0.1	1	56	0.8	0.375	0.014
A × G × L	0.1	1	56	0.5	0.482	0.009
A × G × R	0.6	3	168	1.5	0.216	0.026

Bonferroni-adjusted comparisons showed that horizontal growers had an overall 1.26-mm greater thickness than average growers (p < 0.001), while measurements at 11 mm were 1.02 mm greater than those at 6 mm (p < 0.001). The D7 location demonstrated greater thickness than the M6 location, with a mean difference of 0.42 mm (p < 0.001) (Table 5).

**Table 5:** Bonferroni-adjusted post hoc comparisons of significant effects. Pairwise comparisons were performed using Bonferroni-adjusted post hoc tests following mixed-model analysis of variance. *p < 0.05 was considered statistically significant. HU: Hounsfield units; SE: standard error

Parameter	Comparison	Mean difference	SE	P-value (adjusted)	Cohen's d
Thickness					
Growth (horizontal vs average)	Overall	+1.26 mm	0.08	<0.001*	2.45
Age (adults vs adolescents)	Overall	+0.32 mm	0.06	<0.001*	0.62
Level (11 mm vs 6 mm)	Overall	+1.02 mm	0.08	<0.001*	1.85
Root (D7 vs M6)	Overall	+0.42 mm	0.05	<0.001*	0.85
Height					
Growth (average vs horizontal)	Overall	+0.68 mm	0.08	<0.001*	1.32
Age (adults vs adolescents)	Overall	+0.45 mm	0.07	<0.001*	0.88
Level (7 mm vs 3 mm)	Overall	-7.52 mm	1.12	<0.001*	4.20
Age × level	Adults vs adolescents at 7 mm	+0.55 mm	0.12	0.002*	0.75
	Adults vs adolescents at 3 mm	+0.25 mm	0.12	0.121	0.32
Density					
Age × growth	Adults average vs adolescents average	+97.5 HU	12.4	<0.001*	1.65
	Adults horizontal vs adolescents horizontal	+87.5 HU	12.4	<0.001*	1.45
Growth within adults	Horizontal vs average (adults)	-45.0 HU	12.4	0.003*	0.80
Growth within adolescents	Horizontal vs average (adolescents)	-35.0 HU	12.4	0.082	0.59
Level (11 mm vs 6 mm)	Overall	+90.0 HU	6.5	<0.001*	1.75
Root (D7 vs M6)	Overall	+35.0 HU	5.2	<0.001*	0.65

Buccal shelf bone height

The vertical buccal shelf bone height progressively decreased from the 3-mm to 7-mm assessment level across all groups. The average growers demonstrated greater vertical bone height than horizontal growers at each assessment level. At the 7-mm level, the measured values ranged from 13.8 ± 1.4 to 15.3 ± 1.6 mm in adults and from 13.4 ± 1.4 to 14.8 ± 1.5 mm in adolescents (Table 6).

**Table 6:** Buccal shelf vertical bone height according to assessment level and root location. Data are presented as mean ± SD in millimeters. Vertical bone height was measured at 3, 5, and 7 mm from the cementoenamel junction at the mesial and distal roots of the mandibular first and second molars. M6: mesial root of the mandibular first molar; D6: distal root of the mandibular first molar; M7: mesial root of the mandibular second molar; D7: distal root of the mandibular second molar; SD: standard deviation

Assessment point	Root level	Adult average, n=15	Adult horizontal, n=15	Adolescent average, n=15	Adolescent horizontal, n=15
		Mean ± SD	Mean ± SD	Mean ± SD	Mean ± SD
3 mm	M6	21.6 ± 1.4	21.2 ± 1.3	21.3 ± 1.4	21.9 ± 1.3
	D6	21.9 ± 1.4	21.4 ± 1.3	21.6 ± 1.4	21.1 ± 1.3
	M7	21.0 ± 1.4	21.5 ± 1.4	21.7 ± 1.4	21.2 ± 1.3
	D7	21.2 ± 1.5	21.7 ± 1.4	21.9 ± 1.4	21.4 ± 1.3
5 mm	M6	16.4 ± 1.6	15.5 ± 1.5	15.8 ± 1.6	14.9 ± 1.5
	D6	16.9 ± 1.6	15.9 ± 1.5	16.2 ± 1.6	15.3 ± 1.5
	M7	17.1 ± 1.6	16.1 ± 1.6	16.4 ± 1.6	15.5 ± 1.5
	D7	17.4 ± 1.7	16.4 ± 1.6	16.7 ± 1.6	15.8 ± 1.6
7 mm	M6	14.4 ± 1.5	13.8 ± 1.4	14.0 ± 1.5	13.4 ± 1.4
	D6	14.8 ± 1.5	14.1 ± 1.4	14.4 ± 1.5	13.7 ± 1.4
	M7	15.0 ± 1.5	14.3 ± 1.5	14.6 ± 1.5	13.9 ± 1.4
	D7	15.3 ± 1.6	14.6 ± 1.5	14.8 ± 1.5	14.1 ± 1.5

Mixed-model ANOVA demonstrated significant main effects of age group, growth pattern, root location, and measurement level, all with p < 0.001. Significant root × level (p = 0.001) and age × level (p = 0.015) interactions were also observed. The growth-pattern × level interaction was not significant (p = 0.305) (Table 7).

**Table 7:** Mixed-model analysis of variance for buccal shelf vertical bone height. Mixed-model analysis of variance was used, with age group and growth pattern as between-subject factors and root location and assessment level as within-subject factors. *p < 0.05 was considered statistically significant. A: age group; G: growth pattern; R: root location; L: assessment level; SS: sum of squares; df: degrees of freedom; ηp²: partial eta squared

Source of variation	SS	df₁	df₂	F‑value	P-value	ηp²
Between subjects						
Age group (A)	16.2	1	56	18.2	<0.001*	0.245
Growth pattern (G)	32.0	1	56	36.0	<0.001*	0.391
A × G	0.4	1	56	0.5	0.482	0.009
Within subjects						
Root (R)	14.5	3	168	12.1	<0.001*	0.178
Level (L) - (3, 5, 7 mm)	98.3	2	112	52.3	<0.001*	0.483
R × L	7.6	6	336	3.8	0.001*	0.064
A × L	8.5	2	112	4.5	0.015*	0.074
G × L	2.3	2	112	1.2	0.305	0.021
A × R	2.8	3	168	2.3	0.079	0.039
G × R	1.9	3	168	1.6	0.190	0.028
A × G × L	0.8	2	112	0.4	0.672	0.007

Post hoc analysis demonstrated that average growers had 0.68 mm greater overall bone height than horizontal growers (p < 0.001), whereas adults had 0.45 mm greater height than adolescents (p < 0.001). The difference between adults and adolescents was particularly evident at the 7-mm level (Table 5).

Buccal shelf bone density

The bone density varied according to age, growth pattern, root location, and measurement level. The cumulative density was higher among adults than among adolescents in both growth pattern groups. The cumulative density was 665 ± 45 HU in adult average growers and 620 ± 40 HU in adult horizontal growers, compared with 568 ± 35 HU and 533 ± 38 HU in adolescent average growers and horizontal growers, respectively (Table 8).

**Table 8:** Buccal shelf bone density according to assessment level and root location. Data are presented as mean ± SD in HU. Bone density was measured at 6 and 11 mm from the cementoenamel junction at the mesial and distal roots of the mandibular first and second molars. M6: mesial root of the mandibular first molar; D6: distal root of the mandibular first molar; M7: mesial root of the mandibular second molar; D7: distal root of the mandibular second molar; SD: standard deviation; HU: Hounsfield units

Assessment points	Root level	Adults average, n = 15	Adults horizontal, n = 15	Adolescents average, n = 15	Adolescents horizontal, n = 15
		Mean ± SD	Mean ± SD	Mean ± SD	Mean ± SD
At 6 mm	M6	580 ± 50	540 ± 55	490 ± 45	460 ± 50
	D6	610 ± 55	570 ± 50	520 ± 50	490 ± 50
	M7	620 ± 55	580 ± 55	530 ± 50	500 ± 50
	D7	650 ± 60	610 ± 55	550 ± 50	520 ± 50
At 11 mm	M6	680 ± 55	630 ± 60	580 ± 50	540 ± 55
	D6	710 ± 60	660 ± 60	610 ± 55	570 ± 55
	M7	720 ± 60	670 ± 60	620 ± 55	580 ± 60
	D7	750 ± 65	700 ± 60	640 ± 55	600 ± 60
Cumulative	Overall	665 ± 45	620 ± 40	568 ± 35	533 ± 38

Mixed-model ANOVA demonstrated significant effects of age group (p < 0.001), growth pattern (p < 0.001), root location (p < 0.001), and measurement level (p < 0.001). Importantly, the age × growth-pattern interaction was statistically significant (p = 0.012), indicating that the difference in density between growth patterns varied according to age (Table 9).

**Table 9:** Mixed-model analysis of variance for buccal shelf bone density. Mixed-model analysis of variance was used, with age group and growth pattern as between-subject factors and root location and assessment level as within-subject factors. *p < 0.05 was considered statistically significant. A: age group; G: growth pattern; R: root location; L: assessment level; SS: sum of squares; df: degrees of freedom; ηp²: partial eta squared

Source of variation	SS	df₁	df₂	F‑value	P-value	ηp²
Between subjects						
Age group (A)	298,560	1	56	42.1	<0.001*	0.429
Growth pattern (G)	200,760	1	56	28.3	<0.001*	0.336
A × G	48,240	1	56	6.8	0.012*	0.108
Within subjects						
Root (R)	43,560	3	168	18.5	<0.001*	0.248
Level (L) - (6 vs 11 mm)	118,260	1	56	50.2	<0.001*	0.473
R × L	3,120	3	168	2.1	0.102	0.036
A × L	2,580	1	56	2.2	0.143	0.038
G × L	1,760	1	56	1.5	0.225	0.026
A × R	3,840	3	168	2.7	0.065	0.046
G × R	1,980	3	168	1.8	0.150	0.031

Post hoc analysis showed that adult average growers had 97.5 HU greater density than adolescent average growers (p < 0.001), whereas adult horizontal growers had 87.5 HU greater density than adolescent horizontal growers (p < 0.001). Among adults, horizontal growers demonstrated a 45.0 HU lower density than average growers (p = 0.003). The D7 location also demonstrated a greater density than M6 by 35.0 HU (p < 0.001) (Table 5).

Summary of principal findings

Overall, significant differences were observed in buccal shelf thickness, height, and density according to the principal study variables. The growth pattern exerted a particularly strong effect on bone thickness and height, while age and growth pattern both contributed significantly to bone density. The significant age × growth-pattern interaction for density further indicated that the effect of the growth pattern on bone density was not uniform across age groups.

## Discussion

The present study evaluated the mandibular buccal shelf in relation to age and skeletal growth pattern, with a particular emphasis on bone thickness, vertical bone height, and bone density at different posterior mandibular root locations and measurement levels. Overall, the findings indicate that buccal shelf morphology is not uniform and that clinically relevant differences may occur according to skeletal pattern, age, root location, and the level at which the bone is assessed [4]. These findings are relevant because adequate bone quantity and quality are important determinants of primary stability and safe placement of extra-alveolar orthodontic miniscrews [2].

In the present analysis, horizontal-growth subjects demonstrated greater buccal shelf thickness than average-growth subjects did. This finding is consistent with previous CBCT investigations, suggesting that individuals with a more horizontal or hypodivergent skeletal pattern tend to have greater mandibular buccal shelf dimensions [10,11]. Vargas et al. [10] reported an inverse relationship between the gonial angle and mandibular buccal shelf thickness, indicating that short-faced individuals generally possess a greater buccal shelf thickness than individuals with greater vertical facial dimensions. Similarly, Eto et al. [9] found greater buccal shelf bone thickness in hypodivergent individuals, although the effect was particularly evident in the first molar region, rather than uniformly throughout the mandibular buccal shelf. A possible explanation for this is that differences in mandibular morphology associated with vertical skeletal patterns may influence the orientation and thickness of the posterior alveolar and basal bones. Horizontal-growth subjects may consequently present with a more favorable buccal cortical envelope for extra-alveolar anchorage.

Matias et al. [12] evaluated 45 untreated individuals classified as brachyfacial, mesofacial, or dolichofacial, with 15 participants in each group. On the mandibular buccal shelf, measurements were obtained at the distal root of the mandibular first molar and the mesial and distal roots of the second molar at 4 and 8 mm from the cementoenamel junction. Their findings demonstrated that the influence of facial patterns on buccal shelf thickness is site-specific. Greater bone thickness was observed in brachyfacial individuals at the distal root of the first molar, whereas dolichofacial individuals demonstrated greater bone thickness at the mesial and distal roots of the second molar. The authors therefore concluded that the facial skeletal pattern may influence the availability of suitable bone for extra-alveolar miniscrew placement, although the observed variability was considered unlikely to be clinically meaningful.

An important finding was the progressive increase in buccal shelf thickness toward the posterior region, with the distal root of the mandibular second molar demonstrating the greatest values. This observation is in close agreement with that of Eto et al. [9], who reported a significant posterior increase in buccal shelf bone thickness, with the distal second molar region providing the greatest dimensions. Similar posterior increases have been reported in other CBCT studies evaluating the mandibular buccal shelf for miniscrew placement [3,13,14]. The greater dimensions posteriorly may be related to the increasing availability of basal bone and the changing relationship between the molar roots and buccal cortical plate. From a clinical perspective, this supports the consideration of the distal second molar region when an extra-alveolar anchorage site is required, provided the individual anatomical anatomy and mandibular canal position are carefully evaluated. The greater bone dimensions observed in the distal second molar region may indicate greater available osseous dimensions at this location; however, the present findings should be interpreted as anatomical observations rather than evidence that this region is clinically preferable for miniscrew placement.

The present findings also demonstrated a greater bone thickness at a more apical measurement level. This pattern is biologically and clinically plausible because the posterior mandibular alveolar process generally broadens toward the basal region. Similarly, Eto et al. [9] observed a greater thickness at 11 mm than at 6 mm from the cementoenamel junction. A recent CBCT study comparing adolescents and adults likewise reported that horizontal and vertical buccal shelf dimensions varied with measurement depth and anatomical location, with adults showing greater dimensions at deeper levels [7]. Sreenivasagan and Sivakumar [15] found that buccal shelf bone thickness varied significantly with insertion site and depth, with greater bone availability at deeper levels, particularly around the distobuccal aspect of the second molar, with a maximum total bone thickness of 8 mm at the mesial aspect of the second molar and 12 mm at the distobuccal aspect. These findings emphasize that the apparent suitability of the buccal shelf depends not only on the tooth selected but also on the vertical level at which insertion is planned.

Age-related differences were also observed in the present study, with adults generally demonstrating greater bone thickness and density than adolescents did. This finding may reflect progressive skeletal maturation and age-related changes in cortical mineralization [16]. Recent CBCT evidence comparing adolescents and adults has similarly reported greater buccal shelf dimensions in adults, particularly at deeper measurement levels [7]. However, the relationship between age and buccal shelf morphology is not linear. Eto et al. [9] found relatively limited age-related differences in some regions and reported reduced bone height in the second molar region among individuals older than 40 years. Differences between studies may therefore be related to the age ranges examined, skeletal maturation, dentoalveolar characteristics, measurement definitions, and specific anatomical levels selected.

The observed differences in bone density were clinically significant. Although the difference was statistically significant, its clinical significance for miniscrew stability cannot be established from the present measurements alone. Adults showed greater density than adolescents, whereas horizontal-growth subjects showed lower density than average-growth subjects in the present dataset [17]. Bone density contributes to the mechanical environment surrounding a miniscrew [2,18], although the density values obtained from CBCT should be interpreted cautiously because they may be influenced by scanner characteristics, exposure parameters, reconstruction algorithms, and image artifacts [19,20]. Thus, the density findings are most appropriately interpreted as relative differences within the present imaging dataset rather than as absolute measures of the histological bone mineral density.

Clinical implications

Our findings have several potential clinical implications. The mandibular buccal shelf should not be considered a single homogeneous insertion zone. Miniscrew site assessment may take into account the patient's age and skeletal growth pattern, together with the specific molar and root location and vertical level of insertion. The greater bone dimensions observed posteriorly, particularly around the distal aspect of the second molar, may indicate greater available osseous dimensions for extra-alveolar anchorage. However, because this retrospective anatomical study did not directly evaluate miniscrew placement, stability, success, or failure, these findings cannot establish that the distal second molar region or any other region is clinically safer or superior for miniscrew insertion. Greater bone thickness alone is insufficient to establish clinical safety; the position of the inferior alveolar canal, root proximity, cortical morphology, soft-tissue thickness, insertion angle, and planned miniscrew dimensions should also be considered before clinical placement.

Limitations

This study should also be interpreted in light of several limitations. First, the retrospective design did not permit the establishment of a causal relationship between skeletal growth pattern, age, and buccal shelf morphology. Therefore, these findings should be interpreted as an association. Second, the study was restricted to average and horizontal growth patterns because an adequate number of eligible records was not available for the vertical growth-pattern group. The absence of a vertical group limits the generalizability of the findings across the complete spectrum of vertical skeletal patterns and should be considered an important limitation. Third, the study was conducted on Class I skeletal patterns, which limits the generalizability of the findings across the complete spectrum of sagittal skeletal patterns, such as skeletal Classes II and III. Fourth, CBCT-derived density measurements may be affected by technical factors and should not be considered as directly equivalent to conventional CT-derived bone mineral density. Finally, the retrospective sample was derived from a single institution, which may limit the generalizability to other populations. Future prospective, multicenter studies including adequately powered horizontal, average, and vertical growth-pattern groups would help clarify these relationships and determine their practical significance for miniscrew placement.

## Conclusions

The mandibular buccal shelf demonstrated meaningful variations according to age, skeletal growth pattern, root location, and measurement level. Horizontal-growth subjects generally showed greater buccal thickness, whereas adults demonstrated greater overall bone dimensions and density. The distal second molar region appeared particularly favorable anatomically. These findings may support individualized CBCT-based planning of extra-alveolar anchorage, although the retrospective design precludes causal inference and the absence of an adequately represented vertical growth group limits broader generalization.
